# Maintaining physical activity during refeeding improves body composition, intestinal hyperpermeability and behavior in anorectic mice

**DOI:** 10.1038/srep21887

**Published:** 2016-02-24

**Authors:** Najate Achamrah, Séverine Nobis, Jonathan Breton, Pierre Jésus, Liliana Belmonte, Brigitte Maurer, Romain Legrand, Christine Bôle-Feysot, Jean Luc do Rego, Alexis Goichon, Jean Claude do Rego, Pierre Déchelotte, Sergueï O Fetissov, Sophie Claeyssens, Moïse Coëffier

**Affiliations:** 1 Normandie Univ, INSERM Unit 1073, UR, France; 2 Institute for Research and Innovation in Biomedicine (IRIB), University of Rouen, France; 3 Rouen University Hospital, Nutrition unit, Rouen, France; 4 Rouen University Hospital, Laboratory of Medical Biochemistry, Rouen, France; 5 Animal Behavior Platform SCAC, University of Rouen, France

## Abstract

A role of gut-brain axis emerges in the pathophysiology of anorexia nervosa and maintaining adapted physical activity during refeeding remains discussed. We aimed to assess gastrointestinal protein metabolism and investigate the contribution of physical activity during refeeding in C57BL/6 mice with activity-based anorexia (ABA). ABA mice exhibited lower body weight and food intake with increase of lean mass/fat mass ratio and fat oxidation. Colonic permeability was increased in ABA. *Ad libitum* food access was then restored and ABA group was divided into two subgroups, with access to running wheel (ABA-PA) or not (ABA-NPA). After refeeding, fat free mass was completely restored only in ABA-PA. Colonic permeability was enhanced in ABA-NPA. Finally, muscle kynurenine conversion into kynurenic acid was lower in ABA-NPA who also exhibited altered behavior. Maintaining physical activity during refeeding may thus limit colonic hyperpermeability and improve behavior in anorectic mice.

Anorexia nervosa (AN) is an eating disorder characterized by a difficulty to maintain minimal weight^1^ and occurs mainly in adolescence or early adulthood. AN prevalence is between 0.3% to 0.7%^2^ and its physiopathology is multifactorial with biological, psychological and sociocultural factors and stressful life events. Multiple and severe somatic complications are observed during AN, mainly related to undernutrition and psychiatric disorders. Physical hyperactivity is frequently observed in AN patients^3^,^4^. Interestingly, experimental data reported common central pathways for physical hyperactivity and eating disorders^5^. In a recent study, beneficial effects of moderate physical activity on energy metabolism regulation have been reported in a “self-starvation” mice model: a reduced fat oxidation and a preferential use of glucose to compensate chronic energy imbalance^6^. In addition, refeeding resulted in a preferentially visceral fat gain after classical care with bed rest^7^,^8^. It has been suggested that preserving adapted physical activity during refeeding of AN patients should be safe and beneficial for the restoration of body composition and body distribution of fat mass, although it remains still debated^9^. In addition, recent data underlined that physical activity may reduce psychiatric symptoms, i.e. depression, by increasing muscle kynurenine conversion into kynurenic acid that is unable to cross the blood brain barrier^10^. Kynurenine is produced from tryptophan and metabolized along two distinct arms of the pathway with one leading to the production of the neuroprotective kynurenic acid and the other to the neurotoxic quinolinic acid. The balance between these two metabolites is important in health and disease^11^ and alterations of kynurenine metabolism have been described in AN patients^12^.

Intestinal disorders are frequently observed in AN patients, i.e. altered gastric empting, motility, constipation and abdominal pain^13^ and the role of gut brain axis in the pathophysiology of AN now emerges 14. Intestinal protein metabolism plays an important role in the gut homeostasis, as protein turnover approaches 50%/day in human duodenal mucosa^15^,^16^, which is higher than that of other major tissues such as liver or muscle. Previous studies reported that nutritional states^17^,^18^, refeeding^19^, or specific nutrient supplementation^20^,^21^ can affect gut protein metabolism. In addition, physical activity also affects intestinal protein metabolism and intestinal functions, particularly proteolysis^22^ and intestinal permeability^23^. Recently, we observed an enhanced colonic permeability in C57BL/6 mice during activity-based anorexia (ABA) that is used to mimic restrictive anorexia and long-term body weight loss effects^24^. However, there are currently no data regarding the gastrointestinal protein metabolism in ABA model and its modulation by refeeding associated or not to physical activity.

Thus, we aimed to evaluate the effects of maintaining or not physical activity during refeeding in ABA mice.

## Results

From day 1 to day 16, male C57BL/6 mice were divided into four groups: control, CT (n = 16); Limited Food Access, LFA (n = 16); Pair Fed, PF (n = 16) and Activity-Based Anorexia, ABA (n = 24). At day 17, ABA group was divided into two subgroups: ABA with physical activity maintained during refeeding, ABA-PA (n = 8); ABA without physical activity during refeeding ABA-NPA, (n = 8).

### Physical activity

**Figure 1:**
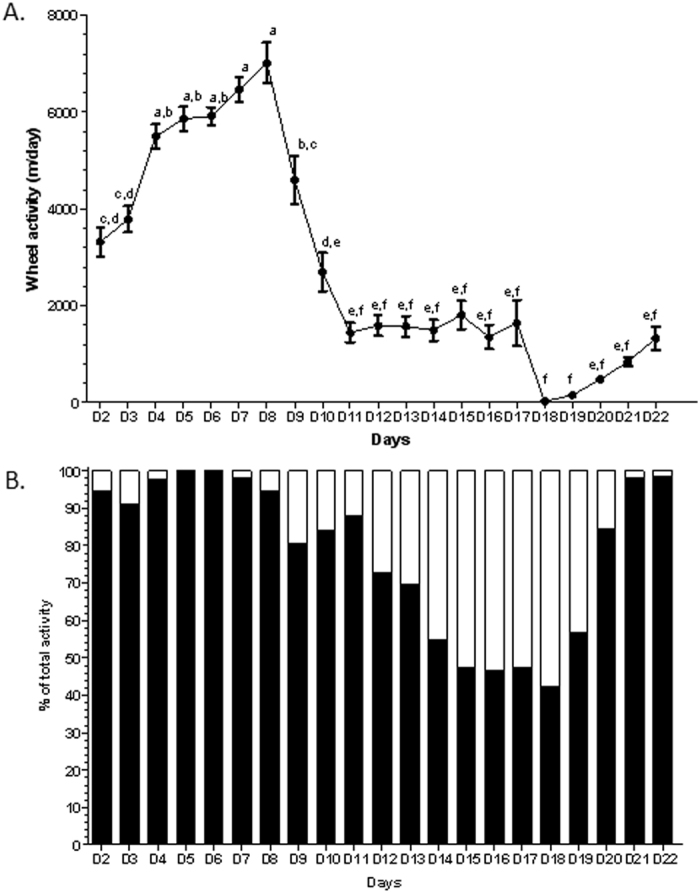
Wheel activity in ABA mice. Wheel activity in mice with activity-based anorexia (ABA). Wheel activity was monitored using RunningWheel® software (Intellibio, France) before (from day 2 to day 5), during limitation of food access (from day 6 to day 17) and after restoration to *ad libitum* access (from day 18 to day 22). (**A**) Total wheel activity, expressed in m/day. (**B**) Contribution of activity from dark (black bars) or light (open bars) phases to total activity expressed in %. Values without a common letter differ significantly, p < 0.05.

Total activity increased during the first days of experiments (Fig. 1A). From day 1 to day 5, wheel activity in ABA mice was predominantly present during the dark phase (Fig. 1B). At the beginning of limited food access, from day 6 to day 8, total activity increased because of increase of activity during both the dark and light phases; from D9, total activity decreased related to a decrease of dark phase activity (Fig. 1A). In addition, a switch of physical activity from dark to light phase was observed in ABA group. After refeeding, a restoration of nycthemeral cycle was observed with an increase of dark phase activity and a decrease of light phase activity (p < 0.05). However, at the end of the study, total wheel activity in ABA mice remained low (Fig. 1A).

### Food intake

**Figure 2:**
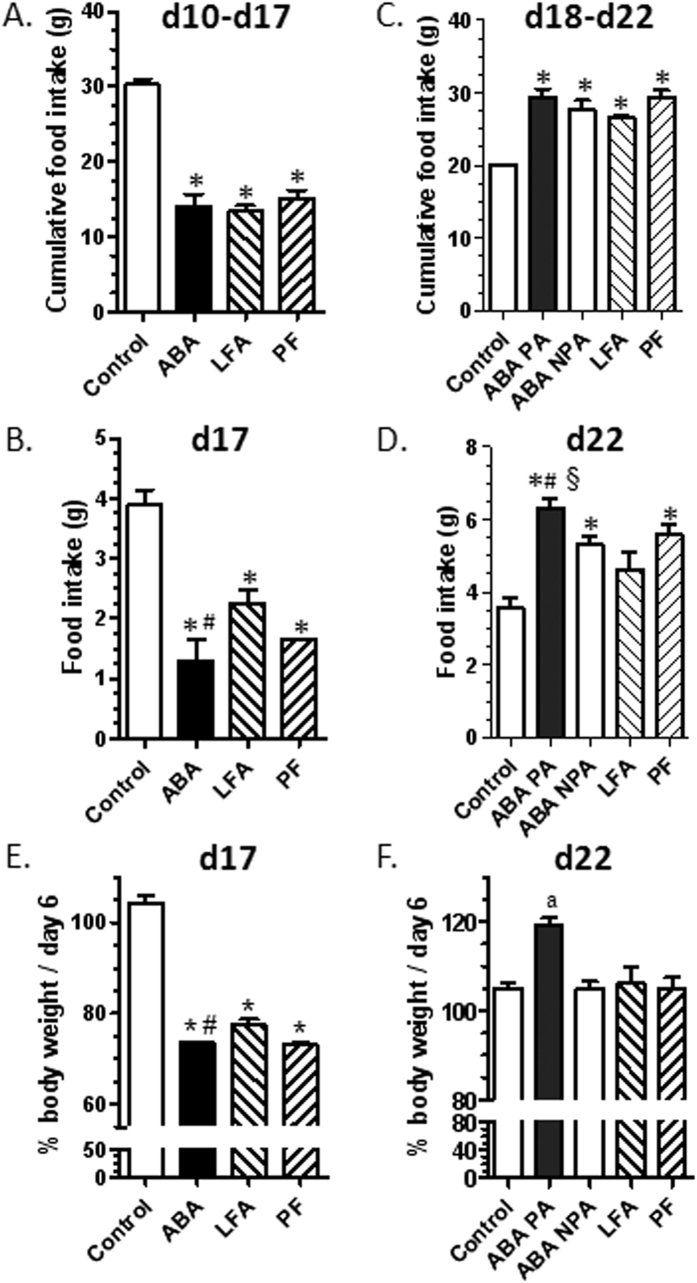
Food intake and body weight. Mice were placed in standard cages with limitation of food access (LFA) or not (control, pair fed (PF) mice), or in cages with activity wheel and limitation of food access (ABA). Progressive limitation of food access started at day 6. Cumulative food intake from day 10 to day 17 (**A**) or food intake measured at day 17 (**B**) in control, ABA, LFA and PF mice. At day 17, *ad libitum* food access was restored and ABA group was divided into two groups, mice with access to running wheel (ABA-PA) or not (ABA-NPA). Cumulative food intake from day 18 to day 22 (**C**) or food intake measured at day 22 (**D**) in control, ABA-PA, ABA-NPA, LFA and PF mice. Body weight changes at day 17 (**E**) and day 22 (**F**) was expressed as % of day 6. *p < 0.05 vs control. ^#^p < 0.05 vs LFA. ^§^p < 0.05 vs ABA-NPA and PF. ^a^p < 0.05 vs other groups.

During the adaptation phase (from day 1 to day 5), there were no significant differences in food intake between ABA, LFA, PF and CT mice. During the limited food access period (from day 10 to day 17), food intake was higher in CT mice than in other groups (p < 0.05, Fig. 2A). No significant differences were observed between ABA, LFA and PF groups until day 16 neither on daily food intake nor on cumulative food intake. At day 17, food intake was lower in ABA than in LFA mice (p < 0.05, Fig. 2B). After refeeding (from day 18 to day 22), food intake was higher in ABA-PA, ABA-NPA, LFA and PF mice than in CT (p < 0.05, Fig. 2C). Moreover, food intake was significantly higher in ABA-PA than in ABA-NPA mice and LFA mice at the end of the protocol (p < 0.05, Fig. 2D).

### Body weight and body composition

**Figure 3:**
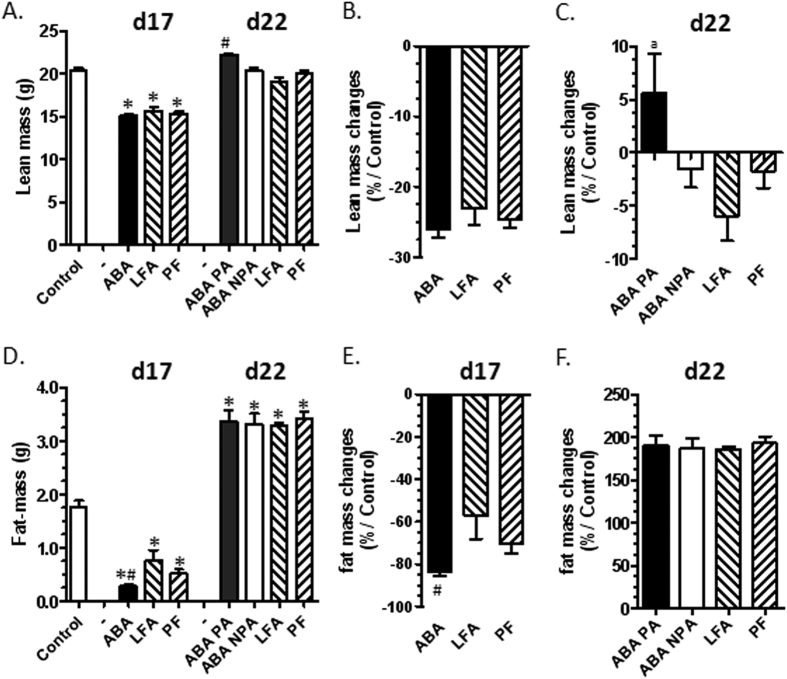
Body composition before and after refeeding. Mice were placed in standard cages with limitation of food access (LFA) or not (control, pair fed (PF) mice), or in cages with activity wheel and limitation of food access (ABA). Progressive limitation of food access started at day 6. At day 17, *ad libitum* food access was restored and ABA group was divided into two groups, mice with access to running wheel (ABA-PA) or not (ABA-NPA). Fat free mass (**A–C**) and fat mass (**D–F**) were measured by EchoMRI. Fat free mass (**A**) and fat mass (**D**) were expressed in g, lean mass changes (**B–C**) and fat mass changes (**E–F**) were expressed as % of control. *p < 0.05 vs control. ^#^p < 0.05 vs LFA. ^a^p < 0.05 vs other groups.

From day 1 to day 5, there were no significant differences in body weight between all the groups. During the limited food access period, body weight decreased in ABA, LFA and PF mice compared with CT (p < 0.05). However, at day 17, weight loss was more pronounced in ABA compared with LFA mice (p < 0.05, Fig. 2E). At day 17, lean mass was similarly reduced in ABA, LFA and PF compared with CT (p < 0.05, Fig. 3A,B). Fat mass was also lower in ABA, LFA and PF compared with CT (p < 0.05, Fig. 3D) but the loss of fat mass was more pronounced in ABA compared with LFA mice (p < 0.05, Fig. 3E). Consequently, the lean mass to fat mass ratio was significantly increased in ABA (76.7 ± 14.2) compared with CT (12.8 ± 1.1) and with LFA and PF mice (35.8 ± 9.3 and 33.1 ± 19.4, respectively).

During refeeding, body weight rapidly increased in LFA, PF, ABA-PA and ABA-NPA (p < 0.05). At the end of the protocol, weight gain was significantly higher in ABA-PA than in ABA-NPA and LFA mice (p < 0.05, Fig. 2F). Lean mass was completely restored in ABA-PA and thus was higher in ABA-PA compared with other groups (p < 0.05, Fig. 3C). Fat mass was markedly increased in all groups after refeeding and thus became higher compared with CT (p < 0.05, Fig. 3F). The lean mass to fat mass ratio became lower in ABA-PA (6.4 ± 0.3), ABA-NPA (6.1 ± 0.3), LFA (5.8 ± 0.1) and PF (5.6 ± 0.2) than CT mice (all, p < 0.05).

### Protein metabolism

**Figure 4:**
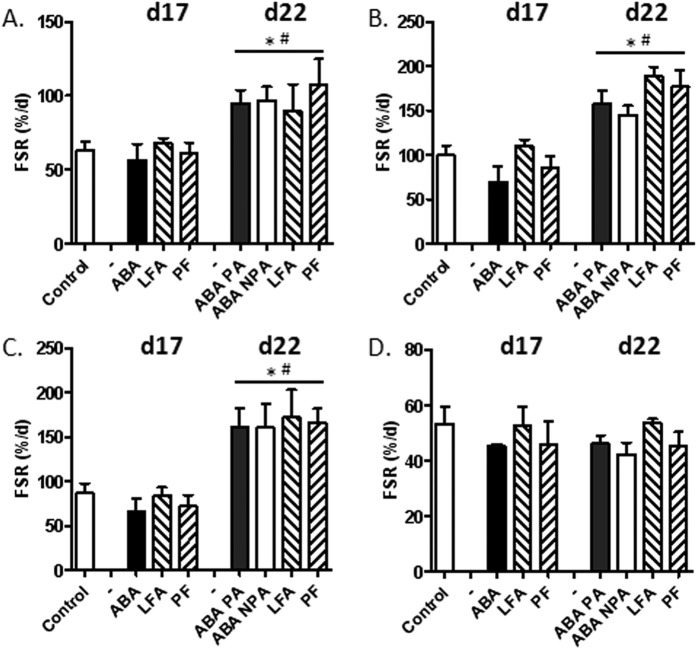
Protein fractional synthesis rate before and after refeeding. Mice were placed in standard cages with limitation of food access (LFA) or not (control, pair fed (PF) mice), or in cages with activity wheel and limitation of food access (ABA). Progressive limitation of food access started at day 6. At day 17, *ad libitum* food access was restored and ABA group was divided into two groups, mice with access to running wheel (ABA-PA) or not (ABA-NPA). Protein fractional synthesis rate (FSR), expressed in %/day, calculated from ^2^H_5_-phenylalanine enrichments in free intracellular amino acid pool and mucosal proteins from gastric (**A**), duodenal (**B**), jejunal (**C**) and colonic (**D**) samples. *p < 0.05 vs control. ^#^p < 0.05 vs day 17.

In preliminary experiments, we assessed the enrichments of ^2^H_5_-phenylalanine in plasma and intracellular free amino acid pools from colonic and jejunal mucosa at different time after ip injection. We observed that enrichment in plasma free amino acid pool remained stable from 5 to 20 minutes and then decreased. Similarly, enrichments in intracellular free amino acid pools from jejunal and colonic mucosa were stable from 5 to 20 minutes (Supplemental Figure S1A). By contrast, the enrichment in jejunal and colonic mucosal proteins linearly increased from 5 to 30 minutes (Supplemental Figure S1 B,C). We thus chose the time of 20 minutes for following experiments. Firstly, in control mice, we observed that mucosal Fractional Synthesis Rate (FSR) was higher in duodenal and jejunal mucosa compared with colonic mucosa (Supplemental Figure S1D) while FSR was not significantly different in gastric mucosa from other tissues. At day 17, mucosal FSR was not affected whatever the tested tissue (stomach, duodenum, jejunum and colon) and the groups, ABA, LFA and PF (Fig. 4). In all groups, refeeding increased gastric, duodenal and jejunal FSR (p < 0.05), but not colonic FSR (Fig. 4D).

Proteasome activities were differentially altered according to the nutritional status (undernutrition at day 17 or refeeding at d22) and the specific studied activity (chymotrypsin-like, trypsin-like, caspase-like or peptidase) as shown in Supplemental table S1. Indeed, it appeared that most of alterations observed at day 17 were restored at day 22. Specifically, we observed that peptidase activity in the duodenal mucosa was markedly increased at day 17 in ABA mice compared with control (p < 0.05) and was completely restored at day 22. However this effect was not shown in LFA and PF mice. Interestingly, we also observed that colonic chymotrypsin-like activity that was reduced in ABA mice at day 17 was markedly increased at day 22 in ABA-NPA mice compared with ABA-PA (p < 0.05, Supplemental Table S1).

### Intestinal permeability

**Figure 5:**
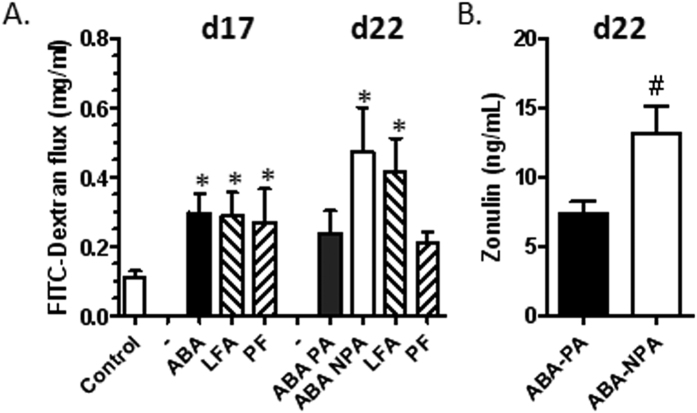
Intestinal permeability before and after refeeding. Mice were placed in standard cages with limitation of food access (LFA) or not (control, pair fed (PF) mice), or in cages with activity wheel and limitation of food access (ABA). Progressive limitation of food access started at day 6. At day 17, *ad libitum* food access was restored and ABA group was divided into two groups, mice with access to running wheel (ABA-PA) or not (ABA-NPA). Colonic permeability (**A**) was assessed by FITC-dextran flux. Zonulin (**B**) was assessed in sera and was expressed as ng/ml. *p < 0.05 vs control. ^#^p < 0.05 vs ABA-PA.

At day 17, colonic permeability was increased in ABA, LFA and PF mice compared with CT (p < 0.05, Fig. 5A). After refeeding, colonic permeability remained increased in ABA-PA compared with controls, even if difference did not reach significance, but was significantly enhanced in ABA-NPA (p < 0.05, Fig. 5A). The expression of tight junction proteins, occludin and claudin-1, was not significantly altered in the colon of ABA, LFA and PF mice neither during undernutrition nor after refeeding (Supplemental Figure S2). We only observed a trend to a decrease of occludin in ABA mice at day 17 and in ABA-NPA mice at day 22 (both p < 0.1, Supplemental Figure S2).

To better understand the effects of physical activity during refeeding on body composition and to confirm the effects on colonic permeability, we performed a second experiment in calorimetric cages with ABA-PA and ABA-NPA groups. To confirm enhanced intestinal permeability, we evaluated serum zonulin that has been recently proposed as a marker of intestinal permeability. Serum zonulin concentration was higher in ABA-NPA than in ABA-PA at day 22 (p < 0.05, Fig. 5B).

**Figure 6:**
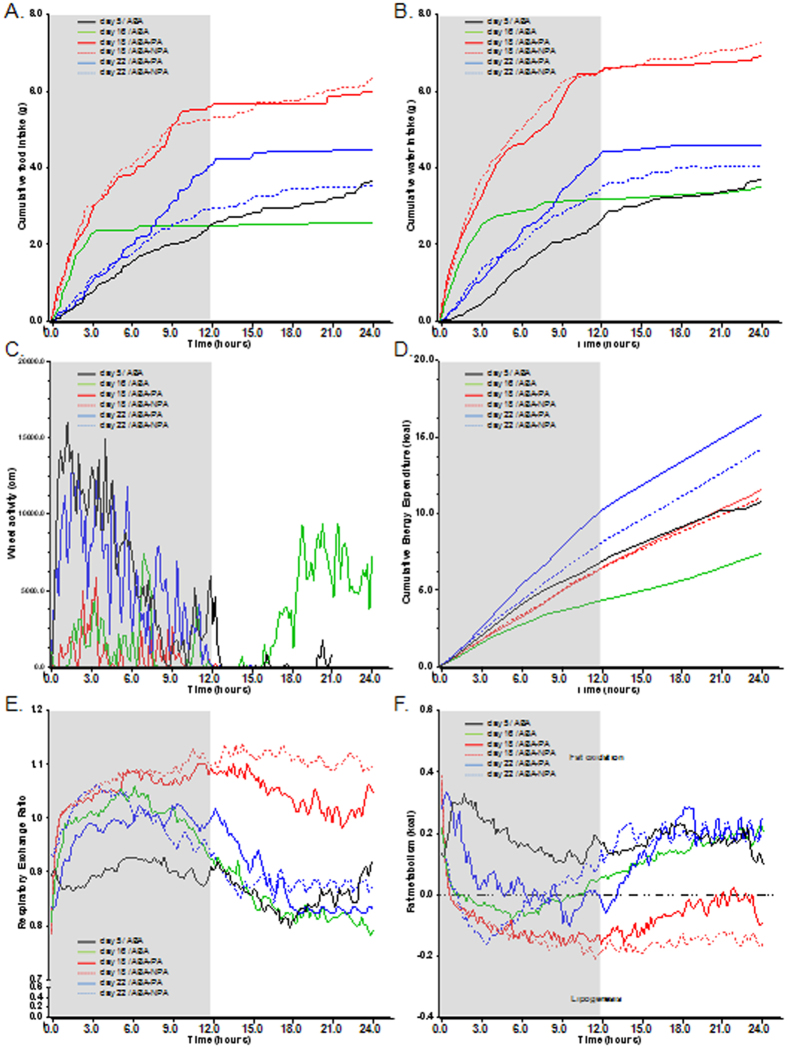
Food and water intakes, wheel activity and metabolism data. Mice were placed in calorimetric cages with activity wheel and limitation of food access (ABA). Progressive limitation of food access started at day 6. At day 17, *ad libitum* food access was restored and ABA group was divided into two groups, mice with access to running wheel (ABA-PA) or not (ABA-NPA). Food intake (**A**), water intake (**B**), wheel activity (**C**), cumulative energy expenditure (**D**), respiratory exchange ratio (**E**) and fat metabolism (**F**) were continuously monitored and calculated. Statistical results are displayed in Table 1.

As previously shown in the first experiment, food, water intake and physical activity patterns were modified in ABA mice (Fig. 6A–C, Table 1).

### Energy metabolism

ABA protocol induced changes in mice energy expenditure (EE) (Fig. 6D, Table 1). At day 16, during the limited food access period, ABA mice had a lower cumulative EE than at day 5 (p < 0.05). During the refeeding period, cumulative EE immediately increased and reached at day 18 the same level that observed at day 5. At the end of the protocol, at day 22, cumulative EE was higher than at day 18. Maintaining physical activity did not affect EE at day 18 but increased EE at day 22 (ABA-PA versus ABA-NPA, p < 0.05).

We then explored whole body metabolism by Respiratory Exchange Ratio (RER) and fat metabolism (fatty acid oxidation and lipogenesis) (Fig. 6E,F). A significant increase of RER was shown during the dark phase of limited food access (day 16) reaching 1.0 which generally represents carbohydrate oxidation. Interestingly, ABA mice showed a progressive decrease of RER during the light phase of limited food access that reached approximately 0.8 suggesting a shift to lipid oxidation. During refeeding, at day 18, RER was maintained around 1.1 both during the dark and light phases suggesting a shift to lipogenesis. Interestingly, lipogenesis was higher in ABA-NPA during the light phase than in ABA-PA (p < 0.05). At the end of the protocol (day 22), we observed the same pattern as observed at day 18 with a shift to lipid oxidation during the light phase, without any difference between ABA-PA and ABA-NPA.

### Locomotor activity

**Figure 7:**
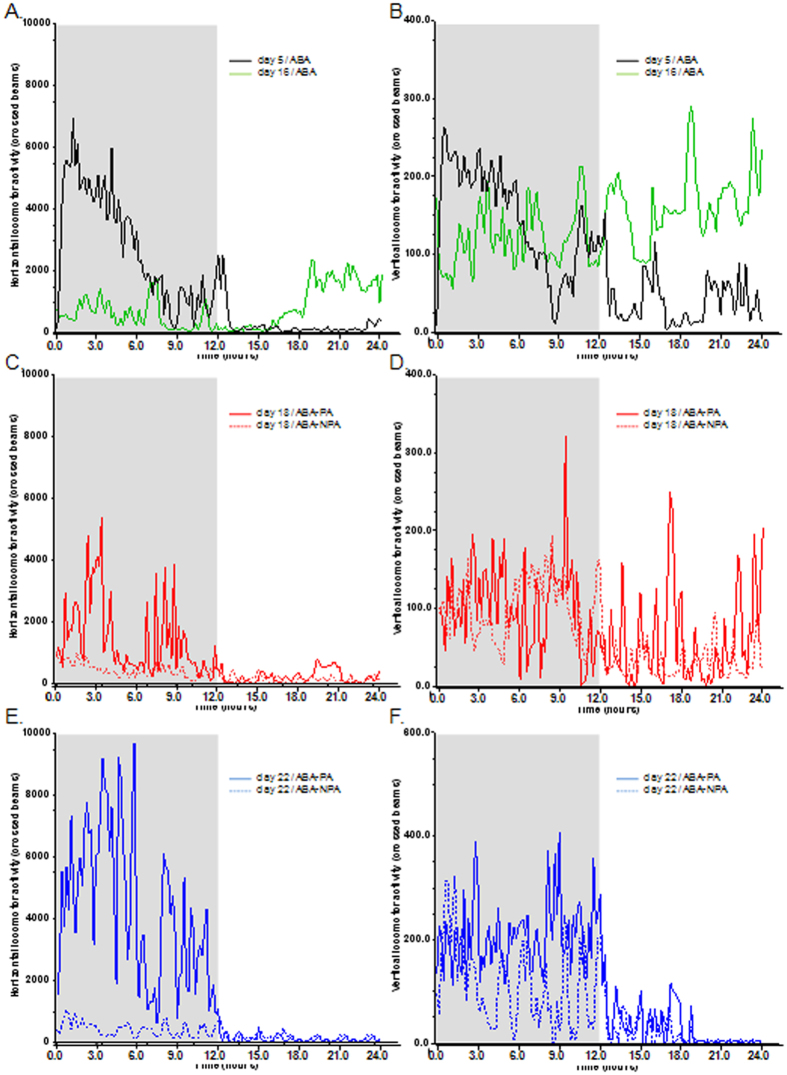
Horizontal and vertical locomotor activities. Mice were placed in calorimetric cages with activity wheel and limitation of food access (ABA). Progressive limitation of food access started at day 6. At day 17, *ad libitum* food access was restored and ABA group was divided into two groups, mice with access to running wheel (ABA-PA) or not (ABA-NPA). Horizontal (**A,C,E**) and vertical (**B,D,F**) locomotor activity were continuously monitored and were expressed as number of crossed beams. Statistical results are displayed in Table 2.

Furthermore, we analyzed ambulatory horizontal and vertical activities. Ambulatory horizontal locomotor activity decreased during the dark phase of limited food access period (p < 0.05, day 16 vs day 5, Fig. 7A, Table 2), while vertical activity increased (p < 0.05, day 16 vs day 5, Fig. 7B, Table 2). During refeeding, horizontal ambulatory activity was restored only in ABA-PA mice, while ABA-NPA exhibits low level of horizontal activity (Fig. 7C,E). Vertical activity was not affected by maintaining or not physical activity (Fig. 7D,F). As these results suggested behavior modifications, we thus performed a third experiment to evaluate mice behavior in dark-light boxes.

### Dark-light Boxes

**Figure 8:**
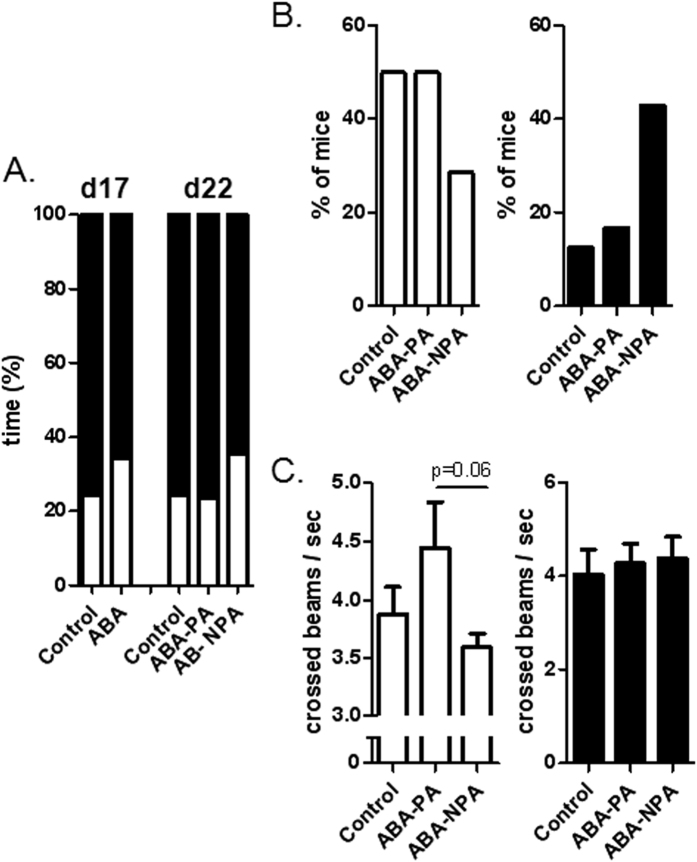
Behavior test. Mice were placed in calorimetric cages with activity wheel and limitation of food access (ABA). Progressive limitation of food access started at day 6. At day 17, *ad libitum* food access was restored and ABA group was divided into two groups, mice with access to running wheel (ABA-PA) or not (ABA-NPA). Mice were then placed in dark-light boxes to evaluate behavior. (**A**) Percent of time spent in each side at day 17 and day 22. (**B**) Percent of mice exhibited zero vertical crossed beams at day 22. (**C**) Number of horizontal crossed beams per second measured at day 22. Open bars represent data obtained in light box and black bars, data obtained in dark box.

At day 17, ABA mice spent more time in the light compartment compared with CT mice (Fig. 8A). After refeeding, at day 22, ABA-NPA mice still exhibited behavior alterations while ABA-PA mice spent the same time in the dark and light compartments compared with CT mice (Fig. 8A). In addition, at day 22, we observed that the percent of mice having no vertical exploratory behavior was different between ABA-NPA and other groups (CT and ABA-PA), both in light and dark sides (Fig. 8B). Finally, at day 22, the exploratory horizontal behavior was lower in ABA-NPA mice compared with ABA-PA in light box, even if difference did not reach significance (p = 0.06), while it was not different in dark side (Fig. 8C).

### Metabolism of Kynurenine

**Figure 9:**
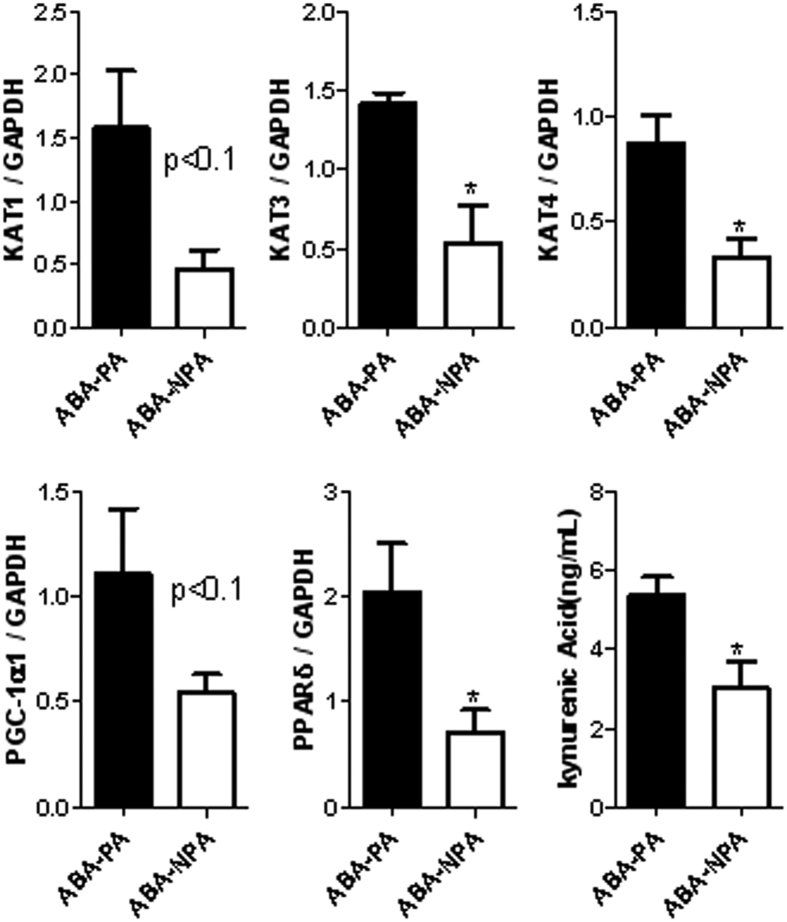
mRNA levels in soleus muscle and serum kynurenic acid level. Mice were placed in calorimetric cages with activity wheel and limitation of food access (ABA). Progressive limitation of food access started at day 6. At day 17, *ad libitum* food access was restored and ABA group was divided into two groups, mice with access to running wheel (ABA-PA) or not (ABA-NPA). Kynurenine aminotransferase (KAT) enzymes, PGC1α, PPAR α and δ mRNA levels were assessed in soleus muscle by RT-qPCR. Kynurenic acid was assessed in sera and was expressed as ng/ml. *p < 0.05 vs ABA-PA.

To better understand the low level of locomotor activity and behavior alterations in ABA-NPA mice, we explored the muscle metabolism of kynurenine. In the soleus muscle, Kynurenine AminoTransferase (KAT)-3 and KAT4 mRNA levels were increased in ABA-PA compared with ABA-NPA mice (Fig. 9, p < 0.05). We also observed a trend for KAT1 (p < 0.1). KAT2 mRNA remained undetectable. Interestingly, physical activity tended to increase PGC1α (p < 0.1) and increased PPARδ (p < 0.05) mRNA expression. PPARα mRNA remained unaffected. In the anterior tibialis, we did not observe any difference between ABA-PA and ABA-NPA mice. We then checked serum kynurenic acid level as a marker of kynurenine metabolism. ABA-PA mice exhibited higher level of serum kynurenic acid (p < 0.05, Fig. 9).

## Discussion

In the present study, we showed (i) that activity-based anorexia model is reproducible in male C57bl/6 mice leading to a lower food intake and a body weight loss, as previously reported^24^,^25^, (ii) that undernutrition led to an increased colonic permeability without modification of protein synthesis in the gastrointestinal mucosa, (iii) that refeeding induced increased of protein synthesis in gastric and small intestinal mucosa but not in the colonic mucosa and (iv) that maintaining moderate physical activity during refeeding limited colonic hyperpermeability, improved lean mass restoration and behavior.

ABA mice showed a lower food intake, an increased body weight loss and an alteration of lean mass/fat mass ratio compared with LFA mice, suggesting the relevance of the model. In addition, hyperactivity frequently occurs in AN patients. ABA mice exhibited typical changes in physical activity pattern with a nycthemeral cycle switch from dark to light phase of physical activity during limited food access which could be related to a food anticipatory activity, even if corticotrope axis may be involved^25^,^26^. It is now established that physical activity and eating disorders are closely linked either in the central circuits leading to food intake reduction and hyperactivity, or during clinical care of patients^27^. Indeed, maintained physical hyperactivity was a predictive factor of higher rate of AN relapse^28^. However, moderate and adapted physical activity has been proposed to be beneficial during refeeding to improve lean mass, bone mineral density or cognitive disorders in AN patients^29^,^30^. We thus evaluated the effects of refeeding associated or not to physical activity. As already shown in food restrictive model^31^, body weight was rapidly restored in all groups. However, body composition was differentially affected by refeeding according to the presence of running wheel or not in ABA mice. Our data are in accordance with previous study in AN patients showing that, after 8 weeks of treatment including or not a resistance training, body mass, body mass index and fat body mass increased significantly in both exercising group and non-exercising group, with no statistical difference on these parameters^32^. However, lean body mass was significantly increased in the exercising group (4.2 ± 3.4 vs 2.11 ± 1.00 kg, p < 0.05). To better understand the impact of physical activity on energy metabolism in ABA model, we performed a second experiment using calorimetric cages equipped with running wheel. Firstly, we observed specific metabolic adaptations in response to limited food access in ABA mice that are in accordance with data obtained in female mice by Mequinion *et al*.^33^. Indeed, fat oxidation decreased during the dark phase (fed state) but then increased during the light phase (fasting state) in ABA. Calorie restriction has been previously reported to increase whole body fat oxidation rates^34^,^35^ and the level of circulating adiponectin leading to increased fatty acid oxidation and reduced deleterious lipid accumulation in other tissues^36^. In contrast, we observed high RER suggesting lipogenesis during refeeding period, and the “rebound effect” observed with the increase of fat mass. Short-term calorie restriction in old rats was associated to a decrease of fatty acid synthase activity leading to a reduced « rebound effect » upon returning to unrestricted diet^31^. In our study, maintaining physical activity during refeeding reduced RER and thus limited fatty acid synthesis. Exercise has been previously reported to reduce appetite, enhance fat oxidation and insulin sensitivity during the early stages of weight regain in calorie-restricted rats^37^. In addition, the level of physical activity in our study, remains lower after refeeding than control level suggesting that we evaluated the effects of a moderate physical activity. Moreover, we observed that ABA-NPA mice exhibited a low level of horizontal activity in the light box at the end of the protocol with a maintained altered behavior after refeeding compared with ABA-PA. A better characterization of behavior in ABA mice before and after refeeding in the presence or absence of physical activity should be further performed. Our data are in accordance with a recent study reporting anxiety level alterations in adolescent female mice undergoing ABA^38^. In healthy adults, regular and moderate physical activity can reduce anxiety symptoms^30^. In addition, increased gain of lean body mass by resistance training was also associated with a better psychological well-being in AN patients^32^. Modifications of kynurenine metabolism have been recently proposed to explain beneficial effects of physical activity on behavior. Indeed, positive effects of physical activity on depression has also been recently reported by Agudelo *et al*. showing that skeletal muscle PGC-1α1 induced by exercise training changes kynurenine metabolism and protects from stress-induced depression^39^. In the present study, ABA-NPA mice exhibited lower levels of mRNA encoding for KAT enzymes than ABA-PA mice. Accordingly, we also found higher level of serum kynurenic acid in ABA-PA than ABA-NPA. These data suggest that adapted physical activity during refeeding may increase muscle conversion of kynurenine into kynurenic acid which do not cross blood-brain barrier in contrast to kynurenine. It would be of interest to analyze the brain levels of kynurenine in the different groups and determine the expression of markers of neuroinflammation and synaptic plasticity. Whether physical activity may have a beneficial effect on behavior in AN patients, and therefore contribute to a better outcome, needs further investigations.

In the present study, we confirmed the increased colonic permeability that is mainly related to undernutrition. Colonic hyperpermeability may be involved in the pathophysiology of AN and its chronicisation^14^. To better understand the increased permeability, we evaluated gut protein metabolism. Mucosal protein synthesis remained unaffected in undernourished mice (ABA, LFA and PF) whatever the studied tissue, stomach, duodenum, jejunum and colon. Although some studies reported that nutritional states, fasted or fed, can affect gut protein synthesis^17^,^18^,^40^, our results are in accordance with previous data showing that protein synthesis is impaired neither in the gastric and duodenal mucosa of malnourished patients^19^ nor in jejunal mucosa of undernourished rats^41^,^42^. Shorter periods of fasting or low food intake are associated to a reduced protein synthesis both in an *in vitro* model of polarized enterocytes^42^,^43^. Thus, it should be interesting to evaluate whether intestinal mucosal protein synthesis may be affected at the beginning of food restriction through regulations of mTOR and GCN_2_ pathways and then restored at the long term (day 17) by an adaptive response that remains unknown. By contrast, specific proteolytic activities of proteasome were altered at day 17, even if we were not able to conclude on global proteolysis as we only evaluated specific activities. Refeeding was also associated in the gastric, duodenal and jejunal mucosa, but not in the colon, with an increase of protein synthesis whatever the condition, ABA with or without physical activity, LFA and PF. These data are in accordance with increased gastric and duodenal protein synthesis after four weeks of enteral refeeding in severe malnourished patients^19^ and with data showing trophic effects of food or nutrients on small intestinal mucosa^40^,^44^,^45^. Even if colonic mucosal protein synthesis seems to be not affected by refeeding, we showed that maintaining physical activity during refeeding may be protective by limiting the increase of intestinal permeability. Indeed, colonic permeability was higher in ABA mice without access to running wheel than other groups that was confirmed by plasma zonulin recently proposed as a plasma marker of intestinal permeability^46^. The protective effects of physical activity on the gastrointestinal tract by reducing the risk of colon cancer, or in inflammatory bowel diseases to prevent constipation is well known^47^. Regular moderate exercise has anti-inflammatory effects^48^ and increases the expression of anti-inflammatory cytokines^49^. Furthermore, in a recent study, moderate exercise attenuated intestinal barrier dysfunction induced in a repeated restrain stress model in mice^50^. In this latter study, exercise, which consisted to thirty minutes per day of swimming, limited bacterial translocation and preserved intestinal permeability through upregulation of antimicrobial peptides^50^. In contrast, intensive exercise has been associated with increased intestinal permeability^23^. Our results suggest that maintaining moderate physical activity during refeeding may limit colonic hyperpermeability.

In conclusion, undernutrition associated to anorexia does not affect gastrointestinal protein synthesis but increases colonic permeability in mice. Refeeding increases mucosal protein synthesis in stomach, duodenum and jejunum but not in the colon. Maintaining moderate physical activity during refeeding does not affect gastrointestinal mucosal protein synthesis but limits colonic hyperpermeability and improves body composition restoration, through positive effects on energy metabolism adaptation. Maintaining physical activity also improves behavior during refeeding in ABA model by increasing kynurenine conversion into kynurenic acid in muscle. These results should be further confirmed in AN patients.

## Material and Methods

All experimental protocols were approved by the ethical committee for animal experimentation named CENOMEXA (acceptance number: N/05-11-12/28/11-15). In addition, authors confirm that all experiments were performed in accordance with relevant guidelines and regulations (Official Journal of the European Community L 358, 18/12/1986).

### Study design: ABA model

Male C57BL/6 mice (Janvier Labs, Le Genest St Isle, France) were divided into four groups: CT, control *ad libitum* (n = 16); LFA, control with Limited Food Access (n = 16); ABA, with limited food access and running wheel (n = 24) and PF, *Pair Fed* (n = 16). From day 1 to day 5, mice were acclimatized in individual cages at 23 °C with a reversed 12-h light-dark cycle (dark phase: 9:30 AM–9:30 PM) and had free access to water and standard diet. ABA mice were placed in individual cages with an activity wheel with RunningWheel software (Intellibio, France). Wheel activity was continuously recorded. LFA and CT mice were placed in individual cages without activity wheel. Both ABA and LFA had a progressive limited food access from 6h/day (day 6) to 3h/day (day 9) until the end of the experiment, as previously described^51^. From day 6 to the end of experiment, mice in the PFgroup had free access to a similar quantity of food consumed in the ABA group. Food was given at the beginning of the dark phase (9:30 AM) while water remained in free access. Body weight, water and food intake were daily measured at 9:00 AM. In the ABA and LFA groups, water and food intake were also measured when food was removed. If weight loss exceeded 20% in 3 consecutive days, mice were euthanized for ethical reasons. At day 17, *ad libitum* food access was restored and ABA group was divided into two subgroups: ABA-PA, physical activity maintained during refeeding with RunningWheel (n = 8); ABA-NPA, without physical activity during refeeding (n = 8). Experiments were carried out until day 22.

To better understand the impact of physical activity on energy metabolism and body composition in ABA model, we reproduced a second experiment using combined indirect calorimetric cages equipped with running wheel (PhenoMaster, TSE Systems GmbH, Germany). Metabolic cages allowed continuous monitoring of energy expenditure (EE) and metabolic gas exchange, the rate of O_2_ consumed (VO_2_) and CO_2_ produced (VCO_2_) in ml/h. Thus, the respiratory exchange ratio (RER) was measured to estimate the proportion of carbohydrates, lipids and proteins contribution to whole-body energy metabolism of ABA mice: complete reliance on carbohydrate oxidation (RER = 1), protein oxidation (RER = 0,8) or fat oxidation (RER = 0,7)^6^. We have also calculated fatty acid oxidation (FA) using the equation^6^: FA (kcal/h) = EE × ((1-RER)/0,3). Body weight, food and water intakes, running wheel activity were automatically monitored by the system. Home cage horizontal and vertical ambulatory activity of ABA mice was also evaluated using a multidimensional infrared light beam system.

### Body composition

Body composition was assessed before and after refeeding, at days 17 and 22 using EchoMRI EMR-185 (EchoMRI, Houston, TX), a fast nuclear magnetic resonance method for vigil mice^52^.

### Euthanasia and sampling

An intraperitoneal injection of ^2^H_5_-phenylalanine was performed before decapitation for the analysis of fractional protein synthesis rates. Samples of blood were collected, centrifugated (4 °C, 3000 rpm, 15 min) and serum were frozen at −80 °C. Anterior tibialis and soleus muscles were immediately removed and frozen at −80 °C until RT-qPCR analysis. Gastric and Intestinal samples were collected, washed with ice-cold phosphate buffer saline solution PBS (KH PO4 0,2g, KCl 0,2%, Na2HPO4 1,15g, NaCl 8g, H2O qsp 1000ml), immediately frozen in liquid nitrogen and stored at −80 °C until analysis of tight junction proteins by westernblot (colon), of fractional protein synthesis rate (stomach, duodenum, jejunum and colon) and of the proteasome activities (stomach, duodenum, jejunum and colon). Samples of fresh colon were collected to evaluate paracellular permeability in Ussing chambers as we previously reported that colonic permeability, but not jejunal, was increased^24^.

### Evaluation of intestinal permeability

Colonic permeability was assessed by measuring FITC-dextran (4 kDa) flow in Ussing chambers with an exchange surface of 0.07 cm^2^ (Harvard Apparatus, Holliston, United States). FITC-dextran (5 mg/ml) was placed in the mucosal side. After 3 h at 37 °C, medium from the serosal side was removed and stored at −80 °C. The fluorescence level of FITC-dextran (excitation at 485 nm, emission at 535 nm) was measured in 96- well black plate with spectrometer Chameleon V (Hidex, Turku, Finland). Values were converted to concentration (mg/mL) using a standard curve.

As serum Zonulin, (identified as prehaptoglobin-2) is one of the few known physiological mediators of paracellular intestinal permeability, its concentration was assessed^46^. Serum zonulin concentrations were measured by Zonulin ELISA kit (MyBiosource, San Diego, CA) according to the manufacturer’s instructions.

### Evaluation of tight junction proteins by western blot

Total proteins were extracted from samples of colon using 200 μl of lysis buffer (100 μL Buffer A ×2, 2 μL dithiothreitol 100 mM, 50 μL NP40 1%, 1 μL protease inhibitors P8340 1×, 2 μL phosphatase inhibitors P2850 1×X, for H2O 200 μL). After centrifugation at 13000 g for 2 min at 4 °C, the supernatant was collected and stored at −80 °C. Protein levels were determined using Bradford analysis BioPhotometer (Eppendorf, Le Pecq, France). Tight junction proteins expression, occludin and claudin 1 has been determined using primary antibodies: rabbit anti-occludin (#717800, 1:1000 dilution, LifeTechnologies, Saint Aubin, France), rabbit anti-claudin 1 (#913843A, 1:1000 dilution, LifeTechnologies) and anti-β-actine (#PO161, 1:1000, Sigma Aldrich, Saint Quentin Fallavier, France). Proteins were separated on a 4–20% gradient polyacrylamide gel (Biorad, Marnes la Coquette, France) and then transferred to a nitro-cellulose membrane. The membrane was blocked for 1 h at room temperature with 5% nonfat dry milk in Tris-buffered saline (10 mmol/L Tris, pH 8; 150 mmol/L NaCl) plus 0.05% Tween 20 (TBST) followed by overnight incubation at 4 °C with primary antibodies. After being washed in TBST, the membranes were incubated with appropriate secondary antibodies (1:5000 dilution, SantaCruz Biotechnology, Tebu-bio, Le Perray en Yvelines, France) for 1 h at room temperature. After three additional washes, immunocomplexes were revealed using enhanced chemiluminescence detection system (GE Healthcare, Orsay, France). Protein bands were scanned (ImageScanner III; GE Healthcare) and quantified using Image-Quant TL software (GE Healthcare) to determine the ratio tight junction protein/ß-actine.

### Analysis of fractional protein synthesis rate

To evaluate fractional synthesis rate (FSR) of mucosal proteins, we used the technique of stable isotope-labeled amino acid (^2^H_5_-phenylalanine) incorporation after intraperitoneal (ip) injection. We performed preliminary experiments to choose the timing of injection and we used the dose of 1500 μmol/kg as previously determined in rats^42^. Briefly, after ip injection of ^2^H_5_-phenylalanine, we euthanized mice at different times (5, 10, 15, 20, 25 and 30 minutes, n = 5 for each time). At each time, plasma, jejunal mucosa and colonic mucosa samples were taken to analyze ^2^H_5_-phenylalanine enrichments in plasma amino acid, intracellular free amino acid pools and in proteins. Then, in CT, LFA, ABA and PF mice, we performed ip injection of ^2^H_5_-phenylalanine at the selected time (20 min). Gastric, duodenal, jejunal and colonic samples were taken and immediately frozen in liquid nitrogen.

The enrichments of ^2^H_5_-phenylalanine was determined in the plasma and mucosal intracellular free amino acid pools and in the tissue proteins, by gas-chromatography-mass spectrometry (MSD 5975, Agilent Technologies, Palo Alto, CA), using tert-butyldimethylsilyl derivatives as previously reported^17^,^53^. Appropriate standard curves were run simultaneously for determination of the enrichments. Protein synthesis rate was expressed as fractional synthesis rate (FSR) calculated as follows: FSR (%/day) = [(Et-Eo)/Ep] × 1/t × 24 × 100, where Et is the enrichment in tissue protein at time t, in %; Eo is the natural abundance of the labelled amino acid in intestinal mucosal protein, in %; Ep is the enrichment of intracellular free amino acid pool at time t; “t” is the duration of the tracer infusion, in hour(s).

### Evaluation of the proteasome activities

Gastric, duodenum, jejunum and colonic tissues were homogenized in ice-cold lysis buffer that contained 30 mmol tris-HCl/L (pH 7.2), 1 mmol dithiothreitol/L, and 1% Triton X-100 (Sigma-Aldrich) placed on ice for 15 min and centrifuged for 15 min at 4 °C and 12,000 g. Proteasome activities were evaluated as previously described^54^ by using spectrofluorimetry on a microtiter plate fluorometer (Chameleon) with fluorogenic proteasome substrates Suc-LLVY-MCA (PSIII; Calbiochem) for chymotrypsin-like activity, Boc-LSTR-MCA (Sigma Aldrich) for trypsin-like activity, Z-LLE-MCA (Sigma Aldrich) for caspase-like or peptidyl-glutamyl peptide-hydrolyzing activity and Z-LLL-MCA (Sigma-Aldrich) for peptidase activity in the presence or absence of proteasome inhibitor (10 mmol/L MG132; Sigma Aldrich). Thus, proteasome activity was calculated by using the difference between the activity measured in the absence of MG132 proteasome inhibitor and the activity measured in the presence of MG132 proteasome inhibitor.

### RT-q PCR

After reverse transcription of 1.5 μg total RNA into cDNA by using 200 units of SuperScript™ II Reverse Transcriptase (LifeTechnologies) as previously described^54^, qPCR was performed by SYBR™ Green technology on BioRad CFX96 real time PCR system (BioRad Laboratories, Marnes la Coquette, France) in duplicate for each sample as previously described^54^. GAPDH was used as the endogenous reference gene. We used previously described specific primers for kynurenine aminotransferase 1 (KAT1), KAT2, KAT3, KAT4 and PGC1α, PPARα and PPARδ, factors involved in the signaling cascade activated by physical activity^39^.

### Evaluation of serum kynurenic acid

We evaluated serum kynurenic acid by using commercial kit (Hölzel Diagnostika, Köln, Germany) according to the manufacturer’s instructions.

### Dark and light compartment test

The anxiogenic activity was assessed automatically in a computerized dark and light compartment test (Versamax, AccuScan Instruments Inc, Ohio, USA). This test is a pharmacologically validated assay of anxiety in rodents, based on the conflict between the inherent tendency of mice to explore a novel environment and their natural avoidance of brightly lighted open fields. The apparatus consisted of a Plexiglas enclosure divided into two compartments, each measuring 40 × 20 × 30 cm. One compartment was dark (painted in black and covered with a black Plexiglas lid) and the other compartment (not covered) was clear and illuminated by a 75-W light bulb (200 lx intensity at the level of the light compartment), set 50 cm above the floor. The compartments communicated through an opening (W 5 cm, H 5 cm) located at the base, in the middle of the partition wall. Mice, previously isolated (15 min before the experiments) in individual cages in the experiment room at an ambient temperature of 22 + 1 °C, were placed in the black compartment, which was then covered. The total time spent and locomotor activity in each compartment were recorded during both consecutive periods of 5 min each.

### Statistical analysis

Results, expressed as mean ± standard error mean, were compared using Graphpad Prism version 5.0 (Graphpad Software, La Jolla, United States). Inter-individual comparisons between two groups were performed with Student t test, parametric or non-parametric Mann-Whitney test, and ANOVA followed by Tukey post-tests for more than two groups. A P value < 0.05 was considered significant.

## Additional Information

**How to cite this article**: Achamrah, N. *et al*. Maintaining physical activity during refeeding improves body composition, intestinal hyperpermeability and behavior in anorectic mice. *Sci. Rep.* **6**, 21887; doi: 10.1038/srep21887 (2016).

## Supplementary Material

Supplementary Information

## Figures and Tables

**Table 1:** Statistical results for data presented in Figure 6.

		Cumulative food intake	Cumulative water intake	Wheel activity	Cumulative energy expenditure	Respiratory exchange ratio	Fat metabolism
Two-way ANOVA	hours:	< 0.0001	< 0.0001	< 0.0001	< 0.0001	< 0.0001	< 0.0001
	periods:	0.0005	0.3984	0.3777	0.0013	0.0003	0.0009
p values	interaction:	< 0.0001	< 0.0001	< 0.0001	< 0.0001	< 0.0001	< 0.0001
day 5 vs.							
	day 16	(2.5 → 3.5)	(2.3 → 3.6)	(0.5 → 3.6)	(17.8 → 24)	(3.5 → 10.1)	(2.1 → 3.5)
				(20.3 → 21.5)		(23.6 → 24)	
	day 18 (PA)	(2.5 → 24)	(5.0 → 24)	(1.1 → 1.8)	ns	(11.3 → 24)	(0.7 → 3.8)
							(9.1 → 11.0)
	day 18 (NPA)	(2.5 → 24)	(10.2 → 24)	na	ns	(9.8 → 21.8)	(0.7 → 6.7)
							(9.1 → 12.8)
							(17.3 → 20.1)
	day 22 (PA)	(9.5 → 24)	ns	ns	(16.0 → 24)	ns	(10.5 → 11.7)
	day 22 (NPA)	ns	ns	na	ns	(1.5 → 5.1)	(1.6 → 5.5)
day 16 vs.							
	day 18 (PA)	(13.5 → 24)	(15.5 → 24)	ns	(14.8 → 24)	(16.8 → 24)	ns
	day 18 (NPA)	(12 → 24)	(20.3 → 24)	na	(16.8 → 24)	(14.2 → 24)	(17.5 → 19.3)
	day 22 (PA)	(10.3 → 24)	ns	ns	(9.3 → 24)	ns	ns
	day 22 (NPA)	(18.3 → 24)	ns	na	(12.3 → 24)	ns	ns
day 18 (PA) vs.							
	day 18 (NPA)	ns	ns	na	ns	(17.3 → 18.1)	ns
	day 22 (PA)	(21.3 → 24)	ns	ns	(20.1 → 24)	(22.7 → 24)	(1.1 → 1.3)
	day 22 (NPA)	(11.8 → 24)	ns	na	ns	(17.3 → 24)	(0.6 → 1.3)
day 18 (NPA) vs.							
	day 22 (PA)	(18.7 → 24)	ns	na	ns	(17.3 → 24)	(17.3 → 24)
	day 22 (NPA)	(13.4 → 24)	ns	na	ns	(17.3 → 19.3)	(17.5 → 18.8)
day 22 (PA) vs.							
	day 22 (NPA)	(12.1 → 24)	ns	na	(16.8 → 24)	ns	ns

Data were compared with two-way ANOVA (hours × periods) for repeated measures with Bonferroni post-tests. P values for ANOVA are presented in the 2^nd^ lane. Statistical differences between different periods of the experiments are displayed in the following lanes; (A → B) means significant different from A hours to B hours.

PA, with physical activity; NPA, no physical activity; ns, not significant; na, not applicable.

**Table 2:** Statistical results for data presented in Figure 7.

		Horizontal locomotor activity	Vertical locomotor activity
Two-way ANOVA	hours:	< 0.0001	0.2373
p values	periods:	0.0735	< 0.0001
	interaction:	< 0.0001	< 0.0001
day 5 vs. day 16		(0.3 → 4.6)	(18.6 → 19.8)
day 18 (PA) vs. day 18 (NPA)		ns	ns
day 22 (PA) vs. day 22 (NPA)		(0.8 → 4.0)	ns

Data were compared with two-way ANOVA (hours x periods) for repeated measures with Bonferroni post-tests. P values for ANOVA are presented in the 2^nd^ lane. Statistical differences between different periods of the experiments are displayed in the following lanes; (A → B) means significant different from A hours to B hours.

PA, with physical activity; NPA, no physical activity; ns, not significant.
