# Supplementary material for: Maintaining physical activity during refeeding improves body composition, intestinal hyperpermeability and behavior in anorectic mice
Source: Sci Rep. 2016 Feb 24;6:21887. doi: 10.1038/srep21887 (PMC4764812; doi:10.1038/srep21887)
Supplement: Supplementary Information [file srep21887-s1.doc]

**Maintaining physical activity during refeeding improves body composition, intestinal hyperpermeability and behavior in anorectic mice.**

Najate Achamrah, Séverine Nobis, Jonathan Breton, Pierre Jésus, Liliana Belmonte, Brigitte Maurer, Romain Legrand, Christine Bôle-Feysot, Jean Luc do Rego, Alexis Goichon, Jean Claude do Rego, Pierre Déchelotte, Sergueï O Fetissov, Sophie Claeyssens, Moïse Coëffier

Supplemental Table S1 : Proteasome activities

|  | Chymotrypsin-like | Trypsin-like | Caspase-like | Peptidase |
| --- | --- | --- | --- | --- |
| ***Gastric mucosa*** |  |  |  |  |
| Control | nd | 100.0 ± 43.3 | 100.0 ± 19.1 | 100.0 ± 32.9 |
| ABA (d17) | nd | 64.7 ± 16.7 | 46.2 ± 7.1 * | 98.68 ± 48.1 |
| LFA (d17) | nd | 73.3 ± 19.6 | 30.9 ± 7.1 * | 98.44 ± 29.0 |
| PF (d17) | nd | 90.9 ± 25.2 | 66.76 ± 21.1 * | 95.85 ± 76.7 |
| ABA-PA (d22) | nd | 135.2 ± 45.3 | 160.± 51.9 | 385.1 ± 126.8 |
| ABA-NPA (d22) | nd | 181.9 ± 77.3 | 121.0 ± 37.1 | 339.6 ± 94.8 |
| LFA (d22) | nd | 37.2 ± 36.0 | 106.8 ± 50.7 | 131.4 ± 19.3 |
| PF (d22) | nd | 125.7 ± 36.9 | 109.9 ± 26.8 | 242.0 ± 103.7 |
| ***Duodenal mucosa*** |  |  |  |  |
| Control | 100.0 ± 17.7 | 100.0 ± 21.4 | 100.0 ± 11.7 | 100.0 ± 18.4 |
| ABA (d17) | 54.8 ± 28.7 | 91.2 ± 15.5 | 76.3 ± 9.9 | 272.2 ± 82.7 * |
| LFA (d17) | 94.9 ± 38.4 | 67.2 ± 40.0 | 135.5 ± 23.9 | 95.8 ± 33.8 |
| PF (d17) | 81.1 ± 41.9 | 23.8 ± 17.9 | 139.5 ± 39.4 | 121.0 ± 44.4 |
| ABA-PA (d22) | 69.3± 24.0 | 201.0 ± 64.5 | 72.4 ± 19.4 | 55.9 ± 49.0 ^#^ |
| ABA-NPA (d22) | 122.7 ± 31.3 | 166.1 ± 75.9 | 76.3 ± 17.0 | 77.6 ± 9.9 ^#^ |
| LFA (d22) | 147.8 ± 40.1 | 124.4 ± 12.3 | 89.3 ± 5.2 | 77.3 ± 14.0 |
| PF (d22) | 132.7 ± 31.8 | 238.6 ± 97.3 | 81.3 ± 27.9 | 110.1 ± 33.1 |
| ***Jejunal mucosa*** |  |  |  |  |
| Control | 100.0 ± 38.0 | nd | 100.0 ± 18.1 | 100.0 ± 16.7 |
| ABA (d17) | 58.45 ± 51.0 | nd | 206.7 ± 65.3 | 49.2 ± 37.0 |
| LFA (d17) | 122.7 ± 121.1 | nd | 171.9 ± 97.6 | 84.4 ± 37.0 |
| PF (d17) | 211.3 ± 206.9 | nd | 228.2 ± 65.8 | 62.45 ± 22.4 |
| ABA-PA (d22) | 205.7 ± 63.2 | nd | 90.5 ± 30.9 | 174.1 ± 45.7 |
| ABA-NPA (d22) | 147.9 ± 47.1 | nd | 58.6 ± 11.9 | 119.8 ± 29.4 |
| LFA (d22) | 180.7 ± 95.7 | nd | 73.9 ± 49.9 | 107.7 ± 55.5 |
| PF (d22) | 100.4 ± 48.5 | nd | 59.9 ± 25.7 | 130.2 ± 46.1 |
|  |  |  |  |  |
| ***Table 1 (Continued)*** | | | | |
| ***Colonic mucosa*** |  |  |  |  |
| Control | 100.0 ± 14.7 | 100.0 ± 16.8 | 100.0 ± 13.8 | 100.0 ± 29.1 |
| ABA (d17) | 41.2 ± 12.4 * | 130.9 ± 54.6 | 44.7 ± 5.8 * | 225.3 ± 31.6 * |
| LFA (d17) | 87.75 ± 46.5 | 147.1 ± 38.9 | 62.89 ± 21.6 | 237.6 ± 25.9 * |
| PF (d17) | 29.3 ± 6.9 * | 66.4 ± 19.2 | 38.3 ± 1.8 * | 171.9 ± 36.89 |
| ABA-PA (d22) | 91.6 ± 34.5 | 167.7 ± 58.4 | 148.6 ± 27.39 ^#^ | 74.48 ± 51.5 ^#^ |
| ABA-NPA (d22) | 201.0 ± 44.4 ^#^ | 105.9 ± 12.3 | 141.5 ± 15.62 ^#^ | 85.7 ± 28.3 ^#^ |
| LFA (d22) | 118.8 ± 34.5 | 29.1 ± 15.1 | 123.2 ± 41.7 | 50.0 ± 15.4 ^#^ |
| PF (d22) | 114.7 ± 35.4 | 75.38 ± 7.3 | 231.6 ± 29.0 ^#^ | 110.3 ± 28.2 |

Proteasome activities were expressed as % of control. *, p<0.05 vs Control and #, p<0.05 vs d17.


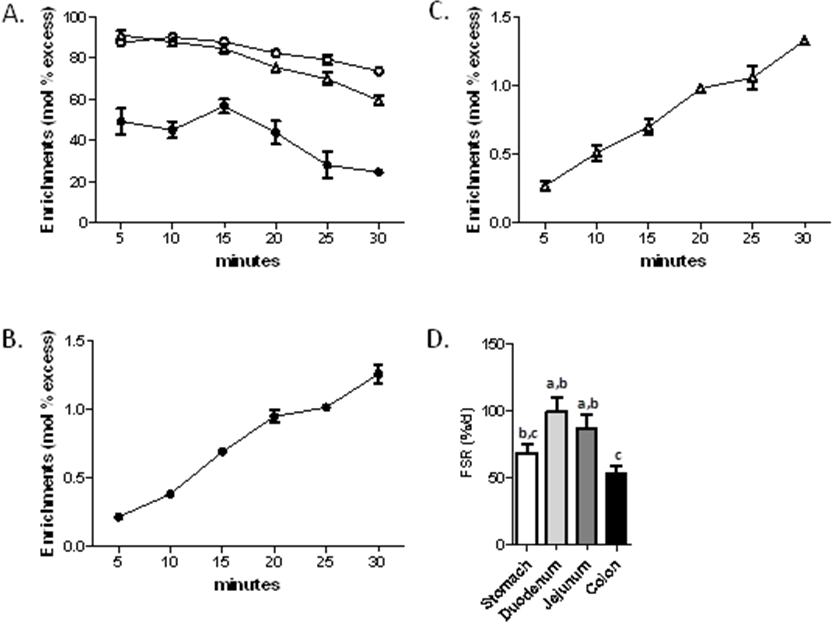


**Supplemental figure S1: ^2^H_5_-phenylalanine enrichment kinetics and Fractional Synthesis Rate**

Enrichment (means ± sem; mol % excess) measured in intratissular free amino acid or plasma precursor pools (A; open circles for plasma, closed circles for jejunum and open triangles for colon) and in mucosal proteins from jejunum (B.) and colon (C.). Mice were injected intraperitoneally with 1,500 µmol/kg of L-[ring-^2^H_5_] phenylalanine and killed after 5, 10, 15, 20, 25 or 30 min (n=5 at each time). (D.) Protein fractional synthesis rate (FSR), expressed in %/day, calculated from ^2^H_5_-phenylalanine enrichments in free intracellular amino acid pool and mucosal proteins from gastric, duodenal, jejunal and colonic samples from control mice. Values without a common letter differ significantly, p<0.05.


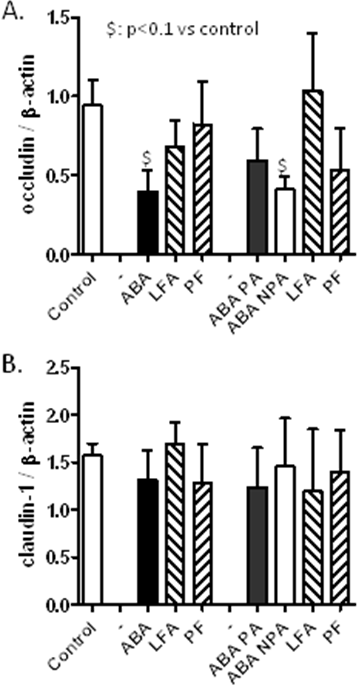


**Supplemental Figure S2: Expression of tight junction proteins, claudin-1 and occludin before and after refeeding.**

Mice were placed in standard cages with limitation of food access (LFA) or not (control, pair fed (PF) mice), or in cages with activity wheel and limitation of food access (ABA). Progressive limitation of food access started at day 6. At day 17, *ad libitum* food access was restored and ABA group was divided into two groups, mice with access to running wheel (ABA-PA) or not (ABA-NPA). Colonic expression of occludin (A) and claudin-1 (B.) was evaluated by western blot.
